# Simulation Safety First: An Imperative

**DOI:** 10.1097/SIH.0000000000000341

**Published:** 2018-11-28

**Authors:** Daniel Raemer, Alexander Hannenberg, Ann Mullen

**Affiliations:** From the Foundation for Healthcare Simulation Safety (D.R., A.H., A.M.), Newton Wellesley Hospital (A.H., A.M.), Ariadne Labs (A.H.), Center for Medical Simulation (D.R.), and Harvard Medical School (D.R.), Boston, MA.

This editorial is simultaneously published in *Simulation in Healthcare, Advances in Simulation,* and *Journal of Surgical Simulation.*

Imagine that the medical and nursing directors of the intensive care unit (ICU) and the director of patient safety had come to you, after an unfortunate clinical incident, with the opportunity to conduct some interprofessional teamwork simulation sessions in the ICU. You arrived with an agreed upon scenario, conquered the logistical barriers, hauled the equipment to the unit, and had been mentally rehearsing an engaging debriefing during a sleepless night. With the agreement of the ICU leadership team, you decided to use only real medications and supplies because this would be more realistic and you generally have a bad feeling about bringing fake things into a patient care area. “What about narcotics, other controlled substances, and expensive or scarce medications?” the nursing director asked. “Oh, let's make an exception for those. We will use prelabeled syringes filled with water,” you had replied quite reasonably.

Later that day, you basked in the glow of a fantastic simulation and debriefing and all of the positive comments of the participants about their experience. The smiles on the leadership's faces had spoken volumes—high fives all around.

Three days hence you are called to the medical director's office where you learn that one of the simulated syringes of fentanyl, authentically labeled, wound up in the scrub jacket pocket of an anesthesiology trainee. He went to the operating room after the ICU scenario and took care of a young child for her surgery. Somehow, he had inadvertently given the tap water “fentanyl” instead of the syringe filled with the real medication he had so dutifully signed out and prepared. The child, probably because of the antibiotic prophylaxis for surgery, has not shown any sequelae so far. The anesthesiology trainee is devastated. The parents of the patient were informed and are upset. Incident reports have been filed, the surgeon is livid, and the chief of anesthesiology has complained to the hospital president. An internal investigation has been initiated. The Drug Enforcement Agency and the Food and Drug Administration are to be informed. How do you feel?

Although this is an imagined scenario, the possibility of simulation-related mishaps resulting in patient, participant, staff, or bystander harm is a real one. A number of incidents have been reported in the literature anecdotally.^1,2^ One such incident has stood out in which simulated intravenous fluid was administered to multiple clinic patients, possibly resulting in the death of one.^3,4^ However, more have been described verbally to us because we have discussed the issue of simulation safety with our colleagues. The hazard seems to be genuine and merits a systematic approach to identifying and mitigating this safety risk of simulation.

Medications and supplies intended for use in the clinical environment and simulated ones intended to be used in the educational setting have potential to become interchanged and used for the wrong purpose. The range of potential mishaps is wide: from the medication errors described above to nursing or medical student practicing injections on each other using unsterile educational supplies. They could be obvious, like mistakenly leaving a liter of simulated intravenous antibiotic behind after an in situ exercise or subtle, such as first-responder participants diverting water-refilled ampules or expired medications to restock their supply bags. They could lead to quite dangerous incidents like the injection into a thumb joint of a simulation participant of 300 μg of epinephrine from an erroneously operated autoinjector to a less serious interchange of a medication past its expiration date.

Such risks are not limited to medications and fluids. Devices modified in some way for simulation and real equipment used in the simulation environment have potential to be confused or exchanged with resulting harm to patients. Defibrillator cables designed for use on mannequins are commonly used and could find their way onto a crash cart intended for patient use. Use of a wall-mounted automated external defibrillator in a facility to use in a simulation could leave clinicians without it when actually needed for a cardiac arrest.

Institutional systems can be misused during simulations that can lead to violations of safe practice. Many resources such as resuscitation teams, extracorporeal membrane oxygenation teams, blood bank, first responders, and uninvolved individuals have been mistakenly called during a simulation exercise. It was reported to us that during a simulation, one participant, unnoticed, called the hospital operator to report a cardiac arrest and invented a room number in another building in the hospital. It took hours to untangle the mess it caused. In another incident, an obstetrician serving as a simulation center director called a colleague for help when faced with two patients arriving in the clinic with rare obstetrical emergencies. The colleague only came reluctantly because she thought the whole thing was a simulation.

A subtle hazard of simulation-based education is that convenient shortcuts taken to implement the simulation efficiently might mislead a learner. For example, not insisting that a learner wear gloves for a line insertion might suggest to them that the practice is unnecessary or, at a minimum, miss an opportunity to model ideal practice. Similarly, a poor medication choice made in a simulation scenario that is not addressed during a debriefing might leave a learner with the impression that the choice was appropriate. We have been told of an instance in which nursing students practiced inserting Foley catheters in a simulation model during class time. Because the focus of the lesson was anatomical and the practice was with a silicone model, the instructor addressed issues of sterility in lecture format. Later, it was discovered that some of the students took the nonsterile Foley catheters home to practice on each other.

Simulation safety risks are not limited to participants. Simulation staff injuries from constantly moving equipment from one location to another have been reported. In one unusual incident reported by a TV news station, property damage ensued when a simulator salesperson left a fully clothed mannequin in his car overnight.^5^ A neighbor called the police to report a frozen elderly person in the passenger seat. The police smashed the windows of the vehicle to gain entrance, only to find that the “person” was plastic.

One recommended approach to conducting safe simulation is initiating a *failure mode effects analysis* (FMEA), an approach borrowed from the safety conscious engineering worlds, for each simulation program to be conducted.^6^ An FMEA attempts to ferret out risks and apply solutions to potential problems before they occur. Implementing an FMEA involves gathering a team responsible for a particular simulation program and brainstorming all of the possible mishaps that could occur and coming up with actions designed to prevent them from happening. Usually, a chart of these “failure modes” and their potential consequences along with the prevention plan is created and can later be used as a basis for safe simulation policies and procedures. The seemingly limitless range of hazards possible suggests that making FMEA a standard part of session planning would be a valuable safeguard.

Various mitigation strategies are available to lessen the probability of identified hazards and should be tested for their effectiveness. One strategy is prominently labeling medication, supplies, and equipment to indicate whether they are not for human use or not. Second, controlling access to simulation supplies, equipment, and spaces can be effective. When a simulation is conducted in or near a clinical environment, using real medications and supplies may be appropriate. Before and after session accounting of medications and supplies, as one does with surgical instruments, could reduce the risk of items going astray. Individual facilities can develop and enforce strict institution-wide policies and procedures to address specific hazards such as protocols for conducting and canceling in situ simulations.^7^ Finally, communicating the risks and policies for safe simulation practice to staff, participants, and others in or near the environment being used is essential. It should be noted that none of these mitigation strategies alone can anticipate all eventualities.

**FIGURE 1:**
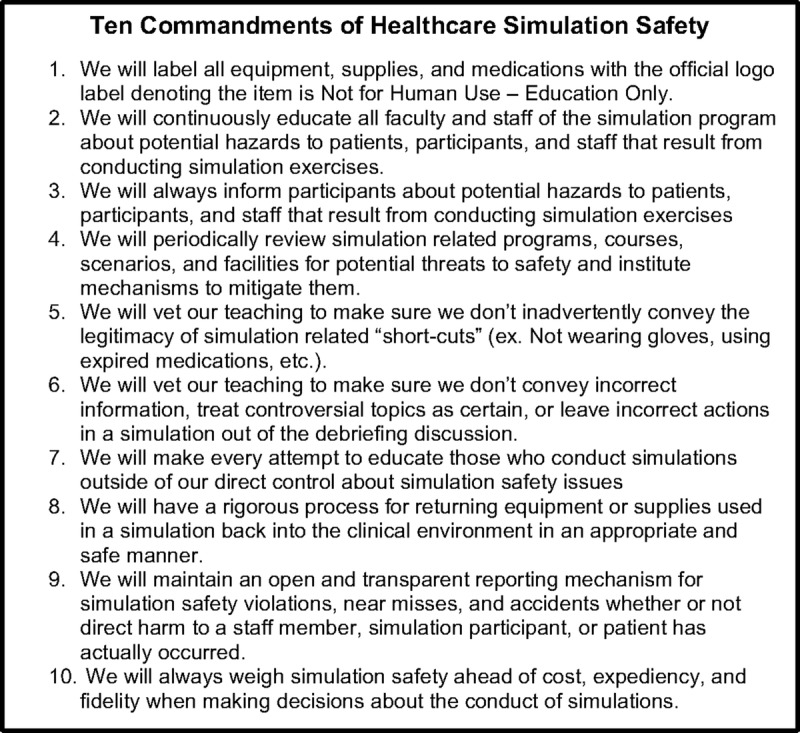
The ten best practices for healthcare simulation safety as listed on the Foundation for Healthcare Simulation Safety website.

**FIGURE 2:**
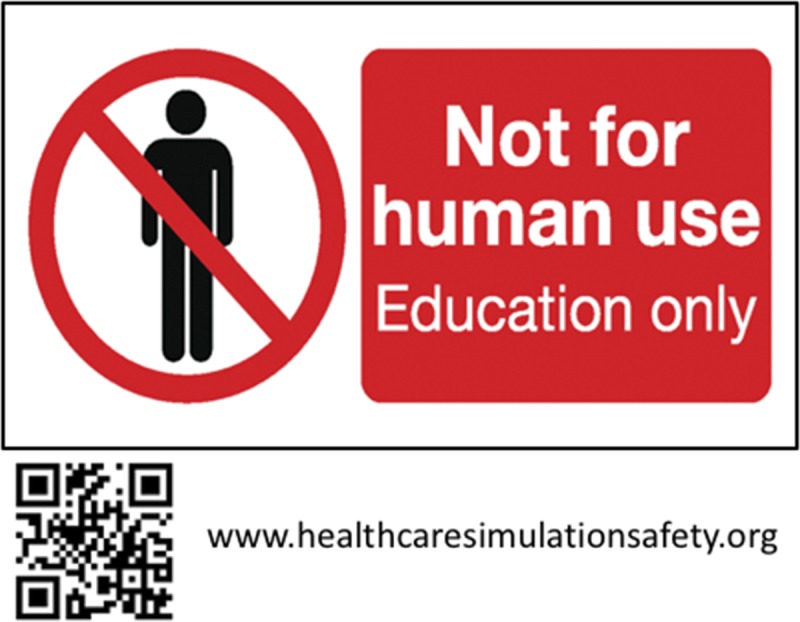
Label for medication, supplies, and equipment used in simulation from the Foundation for Simulation Safety website.

The Foundation for Healthcare Simulation Safety (FHSS), has developed a 10-item “pledge” of “best practices” for simulation programs to adopt to reduce simulation related hazards (Fig. 1). The FHSS is a not-for-profit educational organization recognized as a 501c3 foundation by the US Internal Revenue Service, founded by the authors and supported only by individual philanthropy (http://www.healthcaresimulationsafety.org). The FHSS has been collecting anecdotes of simulation incidents that have or could have resulted in some harm and have been posting them in an anonymous form to help define the scope of the problem in simulation. In addition, FHSS has designed a label to be placed on all simulation medication, supplies, and equipment to identify that it is to be used for educational purposes only (Fig. 2). If adopted universally, it will become a familiar differentiator between real-world and simulation world material.

We urge all personnel involved with simulation education in healthcare to become familiar with potential risks to patients, participants, and staff related to the educational activities they conduct. We urge simulation programs to report incidents to FHSS for posting to the community. In addition, responsible simulation practice demands that mitigation strategies, guided by evidence-based best practices, be adopted to reduce the risk of incidents. Finally, we urge that a universal label be adopted for placement on all medication, supplies, and equipment intended for simulation education use to reduce the chances that they become misused in the real environment.
